# Supplementary material for: Decomposing wealth-based inequalities in ideal cardiovascular health in Kenya
Source: Commun Med (Lond). 2025 Dec 11;6:29. doi: 10.1038/s43856-025-01286-8 (PMC12808186; doi:10.1038/s43856-025-01286-8)
Supplement: Supplementary file 2 — Supplementary Material [file 43856_2025_1286_MOESM2_ESM.pdf]

Decomposing Wealth-based Inequalities in Ideal Cardiovascular Health in Kenya

James Odhiambo Oguta[1*], Penny Breeze[1], Catherine Akoth[1], Elvis Wambiya[1], Grace Mbuthia[2], Peter Otieno[3], Gladwell Gathecha[4], Elizabeth Onyango[4], Yvette Kisaka[4], and Peter J. Dodd[1].

1. Sheffield Centre for Health and Related Research, Division of Population Health, School of Medicine and Population Health, University of Sheffield, Regent Court, 30 Regent Street, Sheffield, S1 4DA, United Kingdom
2. School of Nursing, College of Health Sciences, Jomo Kenyatta University of Agriculture and Technology, P.O. Box 62000- 0200 Nairobi, Kenya
3. African Population and Health Research Center P.O. Box: 10787-00100, Nairobi, Kenya
4. Division of Cancer and Non-Communicable Diseases, Ministry of Health, Afya House, Cathedral Road, Nairobi, Kenya

Corresponding Author*- James Odhiambo Oguta, MSc

Email: mcogutajamo@gmail.com

ORCID: https://orcid.org/0000-0002-2401-9895

Supplementary Material

Supplementary Figures

Multiple Imputation Diagnostics

Supplementary Figure 1: Assessing the pattern of missingness

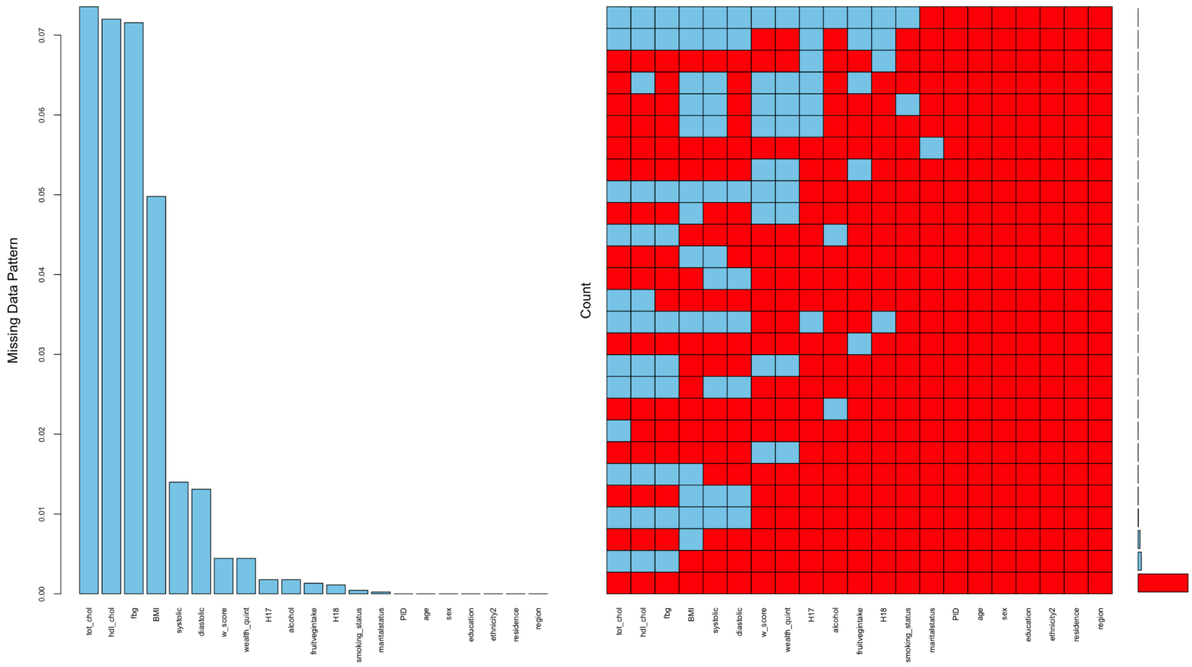

[Legend: Graphs presenting the patterns of missingness of data across key variables. To the left is a plot showing the pattern of missing data across key variables arranged in descending order. The table to the right plots the proportion of the population with complete and missing data by combination of key variables. The graph helps to diagnose the pattern of missingness across key variables to inform the application of multiple imputation. The x-axis contains the key variables that were included in the imputed dataset]

Supplementary Figure 2: Assessing the number of missingness by variable

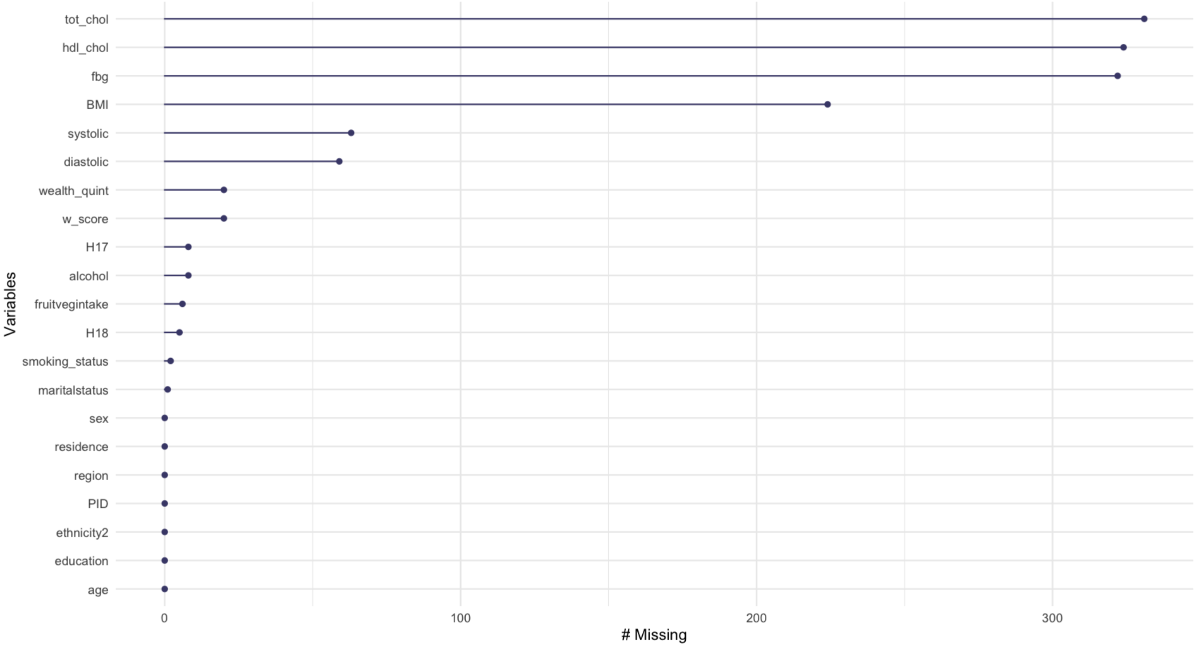

[Legend: This graph presents the counts of missing data for the key variables included in the imputed model. The y- axis contains all the key variables while the x axis has the number of missing observations for each variable.]

Supplementary Figure 3: Assessing the proportion of missingness by sex

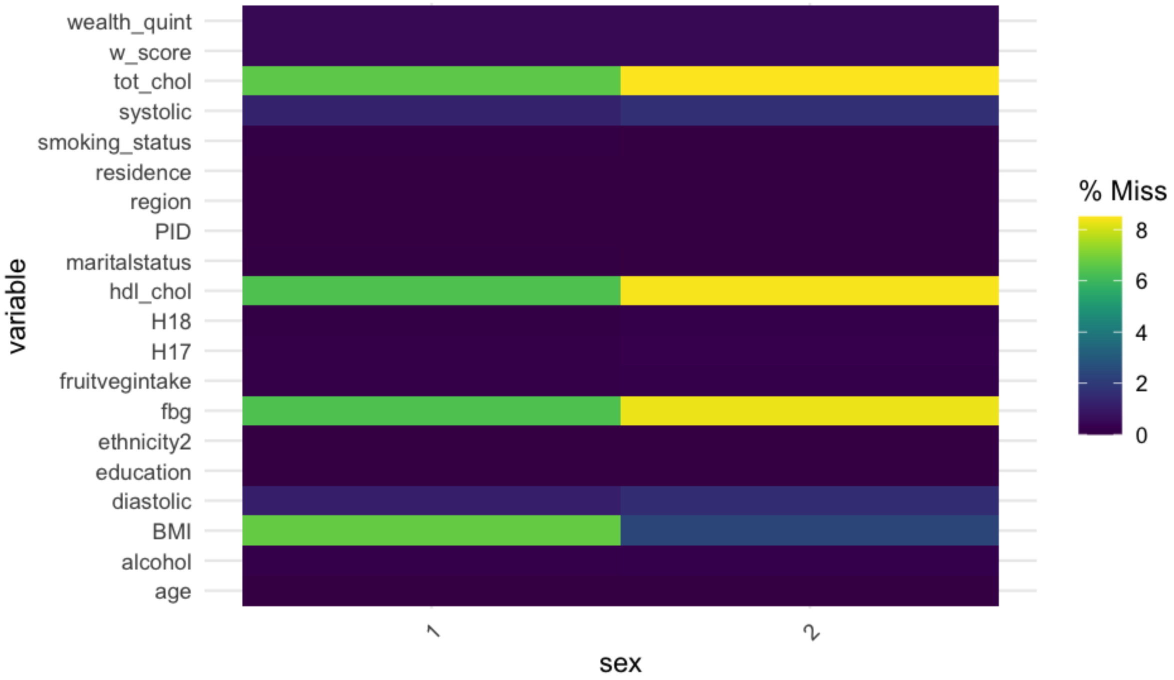

[Legend: This graph presents the percentage of missing variables across key variables by sex. Sex 1 represents females, while Sex 2 represents males. On the Y-axis are variable names as coded in R]

Supplementary Figure 4: Convergence Diagnostics (After 20 imputations)

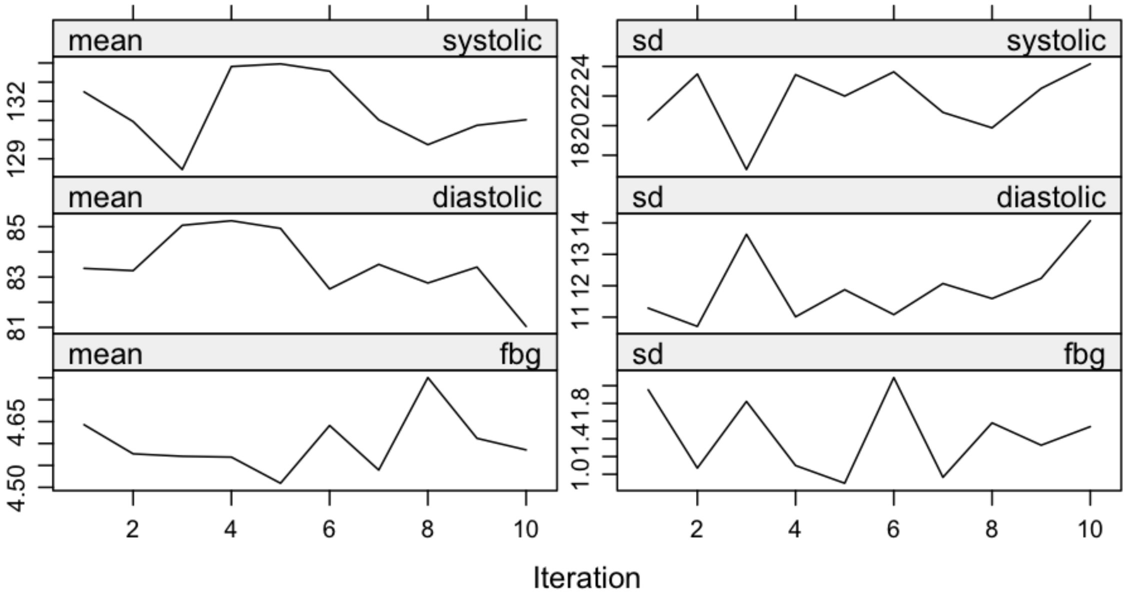

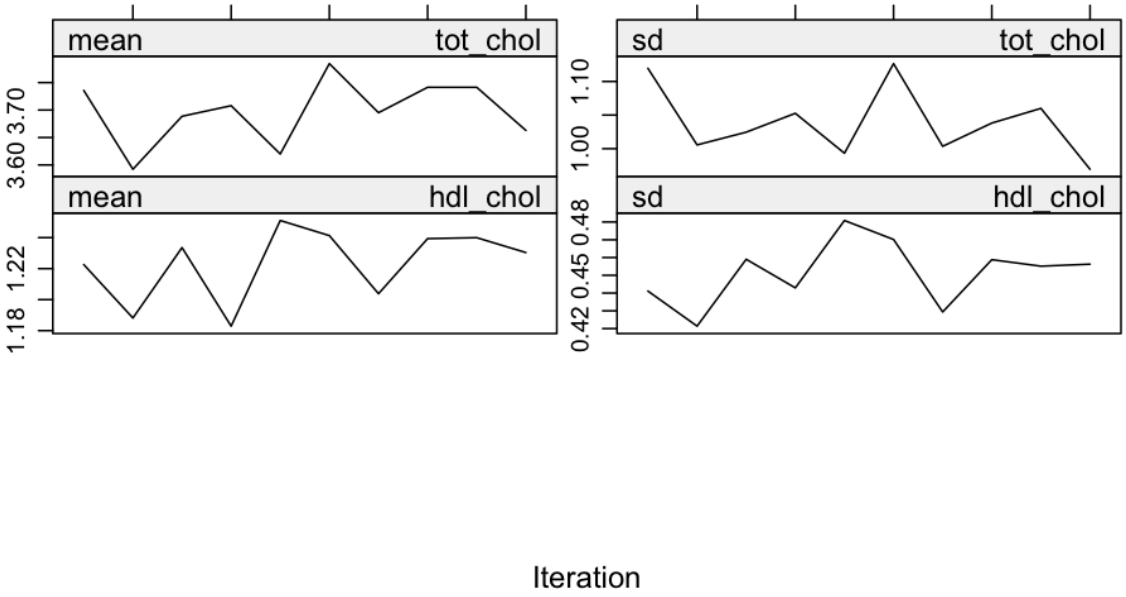

[Legend: These graphs present the diagnostics of imputation model convergence across key variables to assess whether the number of the imputed datasets are adequate. The plots reveal some correlation pattern indicating that 20 imputations are inadequate]

Supplementary Figure 5: Convergence diagnostics (After 80 imputations)

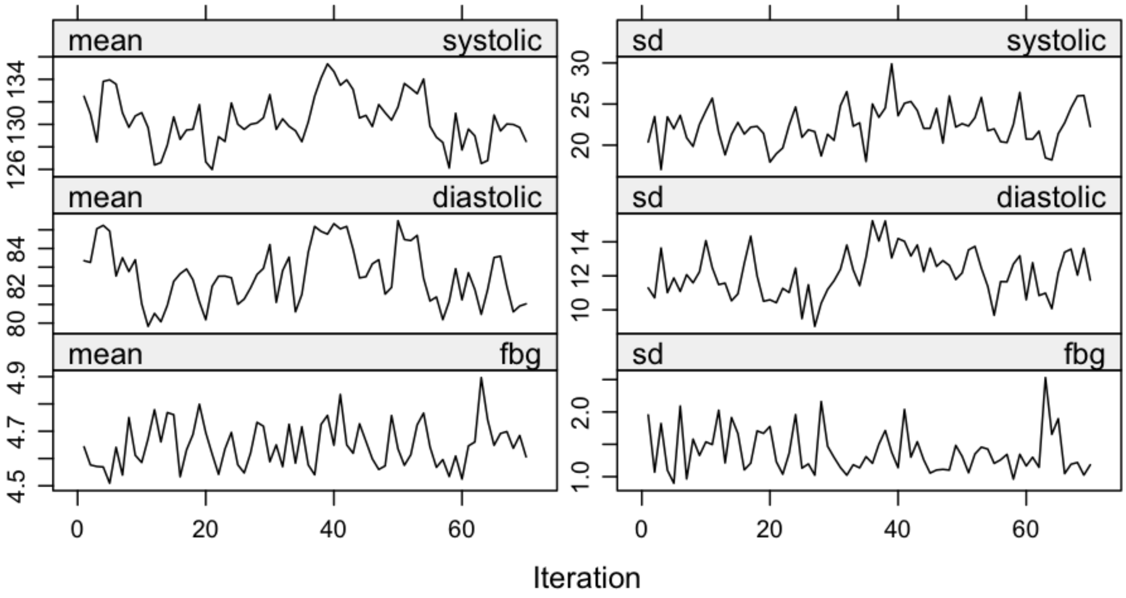

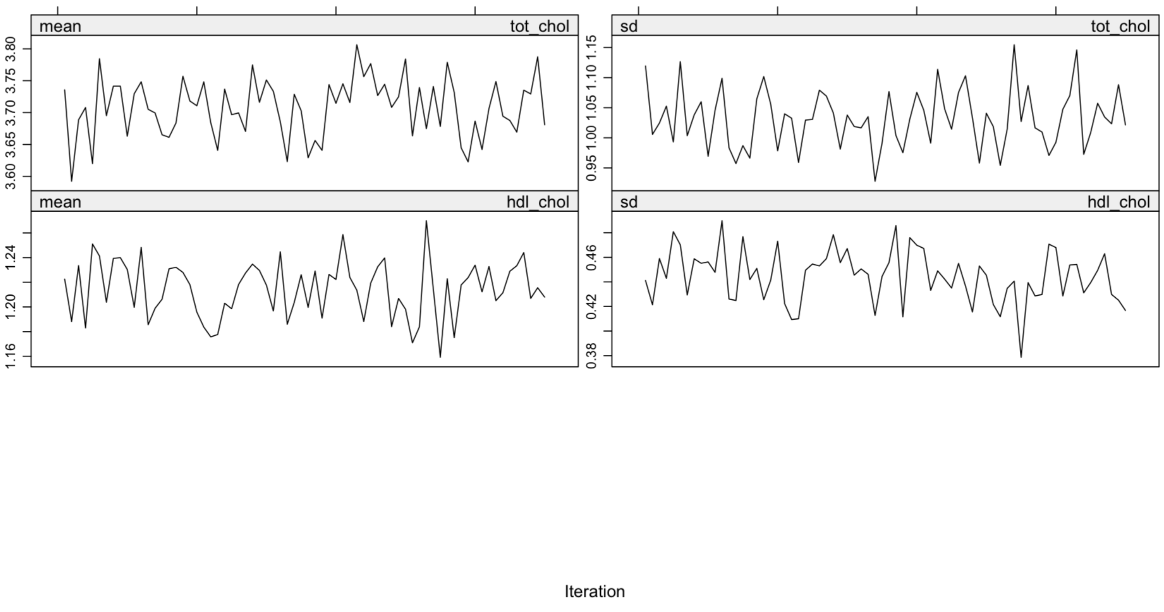

[Legend: These graphs present the diagnostics of imputation model convergence across key variables to assess whether the number of the imputed datasets (80 imputations) are adequate. The plots reveal random pattern indicating that 80 imputations are appropriate]

Supplementary Figure 6: Assessing the distribution of the imputed observations

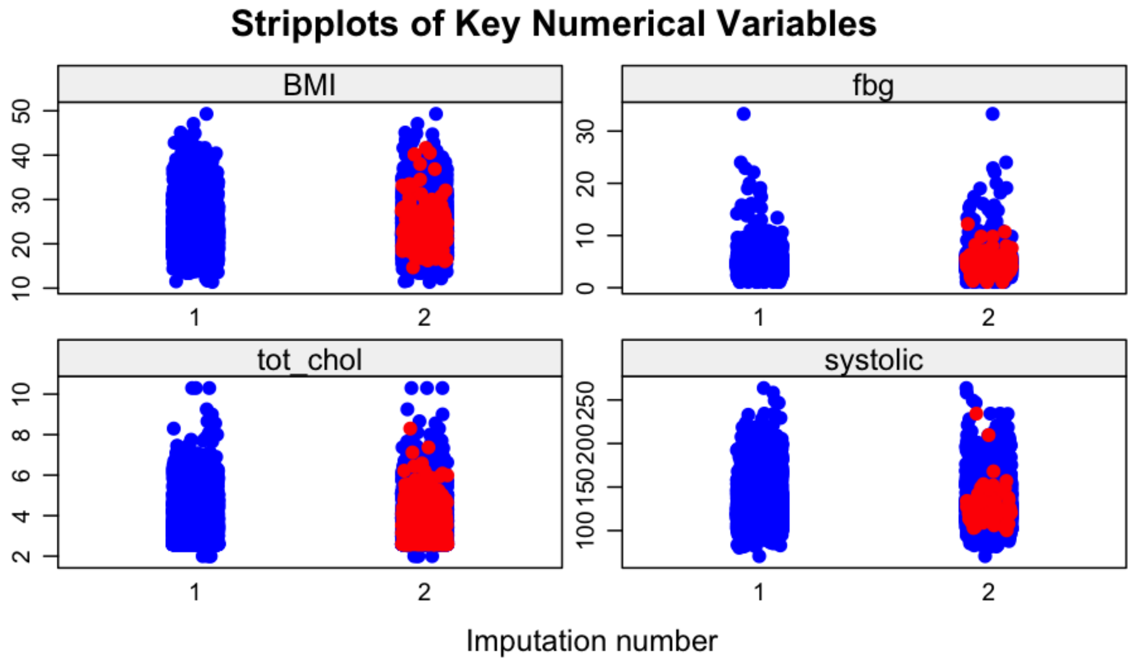

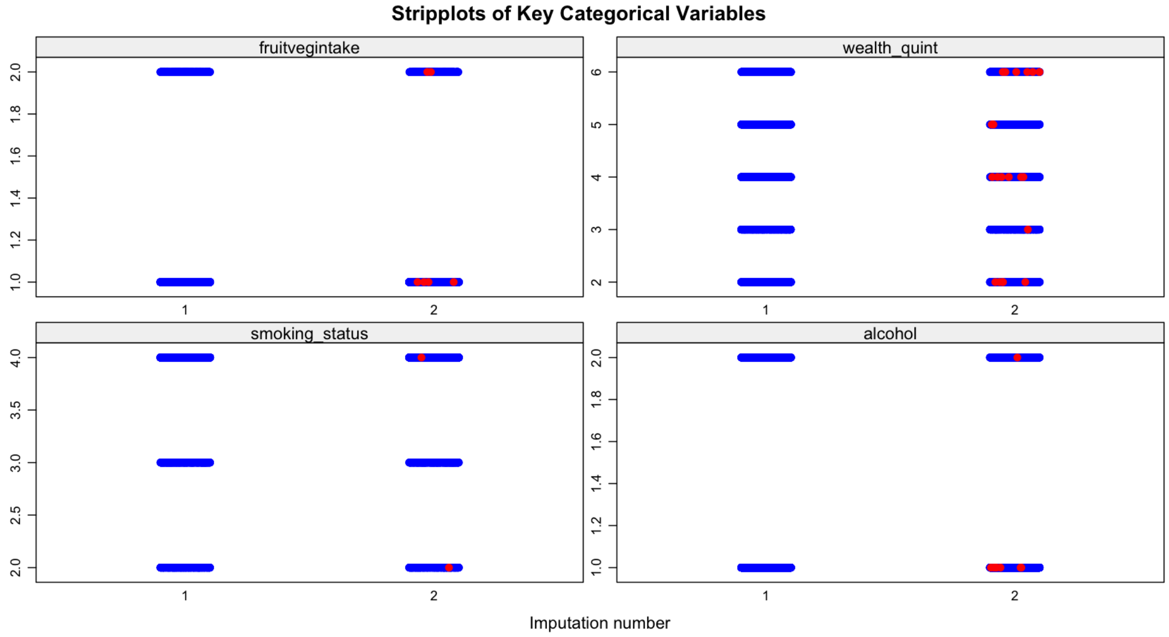

[Legend: These graphs present the strip plots presenting the distribution of original and imputed observations to diagnose whether the imputed data falls within plausible ranges. The blue dots represent observed data points while the red dots represent the imputed observations. All the strip plots show that the imputed observations fall within the ranges of the observed data, which indicates the success of the imputation algorithm.]

Supplementary Tables

Supplementary Table 1: Concentration indices for the socioeconomic inequality in ideal cardiovascular health (CVH) metrics (Complete case analysis-n=3816)

**Ideal CVH Metric**	**Overall**	**Female**	**Male**
**Nicotine exposure**<br>(Never smoked/quit >12 months)	**-0.10 (-0.17, -0.02)***	**-0.45 (-0.65, -0.26)*****	**-0.12 (-0.21, -0.02)***
**Fruit & vegetable intake**<br>(≥5 servings/day)	**-0.09 (-0.18, -0.01)***	-0.08 (-0.19, 0.02)	-0.09 (-0.23, 0.04)
**Physical activity**<br>(>1500 MET-min/week)	0.05 (-0.04, 0.15)	-0.05 (-0.16, 0.05)	**0.22 (0.04, 0.39)***
**BMI**<br>(<25 kg/m^2)	**0.31 (0.25, 0.38)*****	**0.33 (0.25, 0.42)*****	**0.45 (0.31, 0.59)*****
**Blood pressure**<br>(SBP<120 & DBP<80 without treatment)	**0.16 (0.09, 0.22)*****	**0.11 (0.03, 0.19)****	**0.19 (0.09, 0.29)*****
**Fasting blood glucose**<br><5.6 mmol/L (no treatment)	-0.03 (-0.12, 0.06)	0.03 (-0.09, 0.16)	-0.08 (-0.21, 0.05)
**Total cholesterol**<br>(<5.2 mmol/L)	**0.15 (0.04, 0.25)****	0.08 (-0.04, 0.20)	**0.30 (0.11, 0.48)****
**Ideal CVH**<br>(5–7 metrics ideal)	**0.08 (0.02, 0.14)****	**0.11 (0.03, 0.18)****	0.07 (-0.02, 0.17)
Legend: Wagstaff normalisation method used, and standard errors computed using delta method: Bold is statistically significant at p<0.001(***), p<0.01(**), and p<0.05(*). The p-values are from two-sided tests of hypotheses. The overall sample includes 3816 adults composed of 2248 females and 1568 males.			

Supplementary Table 2: Concentration indices for the socioeconomic inequality in poor cardiovascular health (CVH) metrics (Complete case analysis-n=3816)

**CVH Metric**	**Overall**	**Female**	**Male**
Smoking	**0.21 (0.1,0.32)*****	**0.65 (0.53,0.77)*****	**0.21 (0.08,0.33)****
Fruit and vegetable intake	**0.15 (0.08,0.21)*****	**0.16 (0.09,0.24)*****	**0.13 (0.04,0.23)****
Physical Activity	-0.01 (-0.17,0.14)	0.12 (-0.03,0.26)	-0.18 (-0.42,0.07)
Body Mass Index	**-0.34 (-0.42,-0.25)*****	**-0.33 (-0.42,-0.25)*****	**-0.55 (-0.7,-0.39)*****
Blood Pressure	**-0.07 (-0.14,0)***	**-0.09 (-0.17,-0.01)***	-0.06 (-0.16,0.05)
Fasting Blood Glucose	-0.09 (-0.25,0.06)	-0.19 (-0.38,0.01)	0.01 (-0.22,0.25)
Total Cholesterol	**-0.27 (-0.45,-0.1)****	-0.17 (-0.37,0.02)	**-0.52 (-0.78,-0.26)*****
Poor CVH	-0.09 (-0.18,0.01)	-0.09 (-0.2,0.02)	-0.11 (-0.29,0.06)
Legend: Wagstaff normalisation method used, and standard errors computed using delta method: Bold is statistically significant at p<0.001(***), p<0.01(**), and p<0.05(*). The p-values are from two-sided tests of hypotheses. The overall sample includes 3816 adults composed of 2248 females and 1568 males.			

Supplementary Table 3: Concentration indices for the socioeconomic inequality in ideal cardiovascular health (CVH) metrics (Imputed analyses- n=4500)

**CVH Metric**	**Overall**	**Female**	**Male**
Nicotine	**-0.11 (-0.18,-0.04)****	**-0.49 (-0.65,-0.32)*****	**-0.13 (-0.22,-0.05)****
Fruit and vegetable intake	**-0.09 (-0.17,-0.01)***	-0.09 (-0.18,0.01)	-0.07 (-0.2,0.05)
Physical Activity	0.05 (-0.04,0.13)	-0.06 (-0.15,0.02)	**0.23 (0.07,0.4)****
Body Mass Index	**0.32 (0.25,0.38)*****	**0.33 (0.25,0.4)*****	**0.45 (0.32,0.58)*****
Blood Pressure	**0.16 (0.1,0.22)*****	**0.09 (0.02,0.17)***	**0.21 (0.11,0.3)*****
Fasting Blood Glucose	-0.02 (-0.1,0.07)	0.02 (-0.09,0.14)	-0.04 (-0.18,0.1)
Total Cholesterol	**0.14 (0.04,0.23)****	0.08 (-0.03,0.19)	**0.31 (0.13,0.49)****
Ideal CVH	**0.08 (0.03,0.14)****	**0.08 (0.01,0.15)***	0.1 (0.01,0.18)*

Legend: Wagstaff normalisation method used, and standard errors computed using delta method. Bold is statistically significant at p<0.001(***), p<0.01(**), and p<0.05(*). The p-values are from two-sided tests of hypotheses. The overall imputed sample contains 4500 adults composed of 2701 females and 1799 males.

Supplementary Table 4: Concentration indices for the socioeconomic inequality in poor cardiovascular health (CVH) metrics (Imputed analyses- n=4500)

**CVH Metric**	**Overall**	**Female**	**Male**
Nicotine	**0.21 (0.12,0.31)*** **	**0.64 (0.55,0.74)*** **	**0.2 (0.1,0.31)*** **
Fruit and vegetable intake	**0.17 (0.11,0.22)*** **	**0.17 (0.1,0.24)*** **	**0.18 (0.09,0.26)*** **
Physical Activity	0.01 (-0.13,0.15)	0.12 (-0.01,0.24)	-0.17 (-0.4,0.07)
Body Mass Index	**-0.34 (-0.41,-0.26)*** **	**-0.33 (-0.41,-0.25)*** **	**-0.55 (-0.69,-0.4)*** **
Blood Pressure	**-0.07 (-0.13,-0.01)* **	**-0.09 (-0.16,-0.01)* **	-0.07 (-0.17,0.04)
Fasting Blood Glucose	-0.12 (-0.29,0.04)	-0.15 (-0.33,0.03)	-0.12 (-0.41,0.17)
Total Cholesterol	**-0.22 (-0.39,-0.06)** **	-0.14 (-0.32,0.04)	**-0.48 (-0.75,-0.22)*** **
Poor CVH	-0.06 (-0.15,0.03)	-0.04 (-0.14,0.06)	-0.12 (-0.28,0.05)

Legend: Wagstaff normalisation method used, and standard errors computed using delta method. Bold is statistically significant at p<0.001(***), p<0.01(**), and p<0.05(*). The p-values are from two-sided tests of hypotheses. The overall imputed sample contains 4500 adults composed of 2701 females and 1799 males.
